# Ethical reflections on the use of the category “children with disabilities” in quantitative research

**DOI:** 10.3389/fresc.2026.1689272

**Published:** 2026-08-19

**Authors:** Christian Møller-Skau

**Affiliations:** Research Group for Disability and Inclusion, Department of Health, Social and Welfare Studies, University of South-Eastern Norway, Porsgrunn, Norway

**Keywords:** child, dilemma analysis, disability, etichs, quantitative analysis, children with disabilities

## Abstract

Quantitative research plays a vital role in informing public health policies concerning children with disabilities. However, the categorisation of children as ‘disabled’ in statistical analyses raises complex ethical questions. This perspective article offers a conceptual and normative reflection on the ethical implications of such categorisation, drawing on the frameworks of utilitarianism, deontology, and virtue ethics. It argues that while categorisation may be necessary for policy development and resource allocation, it can also perpetuate stigma, discrimination, and social exclusion—particularly when based on inconsistent definitions or overly broad categories. The article contrasts medical and social models of disability and highlights how these frameworks influence who is included within the category “children with disabilities” in research. It further explores how ethical theories can guide researchers in navigating the tension between the need for statistical clarity and the imperative to respect individual dignity and diversity. Special attention is given to intersectionality and to the importance of nuanced, inclusive approaches to data collection. The analysis concludes that categorisation can be ethically justified if conducted with care, transparency, and respect for the lived experiences of children with disabilities. Ethical reflection should be an ongoing process throughout the research lifecycle, not a one-time consideration. This article contributes to the broader discourse on responsible research practices in quantitative studies and calls for ethically grounded methodologies that promote inclusion and equity.

## Introduction

The use of quantitative data is central to research and policy development concerning children with disabilities (CWD). In particular, statistical data are essential for informing public health initiatives and for monitoring the well-being and human rights of this group (1, 2). In such research, however, the social world is necessarily reduced to variables and categories prior to statistical analysis and the identification of associations or effects (3). While this form of categorisation is methodologically necessary, it also introduces methodological (4), ontological (5), and ethical (6) challenges, particularly when applied to vulnerable groups such as CWD, who may face stigma, structural barriers, and limited influence over how they are represented in research (6, 7). Therefore, this perspective article critically examines the ethical implications of categorising children as ‘CWD’ in quantitative research.

While categorisation is applied across disciplines, its meaning and implications differ between fields such as rehabilitation sciences, disability studies, and public health. Regardless of this, across these fields, research involving vulnerable groups requires a particular ethical responsibility to protect participants from stigmatisation or other potential harms throughout the research process (3, 8). In this context, CWD are considered a vulnerable group due to their dual position as minors (< 18 years) and as individuals who may face structural barriers, stigma, and limited opportunities to influence how they are represented in research and statistics (6, 7). To examine this ethical dilemma, three interrelated aspects are important to reflect upon: how disability is defined, how it is measured, and how variation within the group is addressed.

First, the use of socially constructed categories, such as `CWD`, may unintentionally contribute to utter discrimination (2, 6), as disability is a socially and historically contingent concept, shaped by social, political and, institutional contexts (9, 10). In this regard, two influential frameworks are particularly relevant: the medical and the social model of disability. Whereas the medical model locates disability at the individual level, typically based on diagnosis or functional limitations (11, 12), the social model conceptualises disability as arising from societal barriers rather than solely from individual impairments (13).

The second aspect highlights the lack of consensus on how to define and measure CWD in statistics (4, 14). Disability is commonly operationalised through survey instruments such as the Global Limitation Indicator (GALI) (15) or the Washington Group of disability statistics (WG) questions (16). For example, GALI consists of a single question, measured by asking whether a person has been limited in activities people usually do because of health problems for at least six months. In contrast, the WG (16) includes six questions on functional difficulties, such as difficulties in seeing, hearing, walking, remembering, concentrating, self-care, or communicating. As a consequence, these two approaches lead to substantial variation in who is counted as `disabled` in statistics, that is, who is identified as meeting the criteria for `disability` during data handling and conducting statistics. For example, prevalence estimates can vary widely depending on the measurement approach (17), from 8%–15% based on the WG measures (18, 19) to 28% using GALI (20). Over time, such differences may influence not only statistical estimates but also who is regarded as part of the population of children with disabilities (17, 21). These different understandings are also reflected in political debates regarding the normalisation, stigmatisation and marginalisation (9, 22). To summarise, statistical approaches to CWD vary depending on how disability is defined and measured.

Finally, current statistics often fail to reflect the heterogeneity of this group and instead treat CWD as a homogeneous group. Substantial variation within the group is thereby obscured when using dichotomous “disabled”/“not disabled” categories. CWD differ in terms of type and severity of disability, for example, between being blind, deaf, or having intellectual impairments. At the same time, these differences intersect with other social characteristics such as gender, socioeconomic status, and ethnicity. Intersectional theory (23) highlights how such overlapping social positions shape experiences of stigmatisation, normalisation, and marginalisation (24), yet these dimensions are rarely captured in quantitative statistical approaches.

These challenges underline the need for continuous ethical reflection on how survey participants are categorised throughout the research lifecycle. This perspective article critically reflects on the use of the category `CWD` in quantitative studies. Drawing on the ethical theories of deontology, utilitarianism, and virtue ethics, it examines a central ethical dilemma: *the tension between the need to categorise to produce statistical knowledge and the risk that such categorisation may reinforce discrimination and inequality.*

## Methodological approach

This paper adopts a normative ethical approach, grounded in moral philosophy, concerned with evaluating actions and practices in terms of their moral rightness or wrongness, and with articulating standards for how one ought to act (25). This approach is suited to examining ethical issues in research practice, including categorisation, labelling, and potential harm. The analysis draws on ethical theory and existing literature to explore the values and assumptions underlying disability categorisation in quantitative research and their potential impact.

## Ethical research theory

Ethical issues appear throughout the whole research cycle, so addressing them early is necessary. A theory-based approach is well-suited to this purpose (25), as moral philosophers emphasise that it helps ensure that decisions are made by reflecting on ethical principles grounded in moral theory. However, the theory-based approach is criticised for being top-down, in the sense that general ethical theories are applied to specific situations, potentially overlooking contextual and empirical complexities. Ethical theories also have limitations, and no single theory can therefore be “best” or “correct” to use (25). For the purposes of this article, I draw on utilitarianism, deontology, and virtue ethics, which are presented in a research context in the following sections.

## Utilitarianism

Utilitarianism is a philosophical approach that advocates maximising overall good, and the most ethical choice is the one that produces “*the greatest amount of good for the greatest number*” (25). As a consequentialist theory, it emphasises the results of actions when evaluating their moral worth (26). The principle of good in utilitarianism is traditionally associated with happiness, and philosophers John Stuart Mill and Jeremy Bentham described that actions are right when they promote happiness and wrong when they tend to produce the reverse of happiness (25).

Utilitarianism has three main approaches considered relevant. First, *act utilitarianism* focuses solely on actions that yield the maximum happiness for the greatest number of people. It does not consider other factors; hence, a specific individual's preferences are just as significant as anyone else's. This is due to the impartial nature of utilitarianism, where no person's happiness is regarded as more important than others (26). Second, *rule utilitarianism* could offer a contrasting perspective since it proposes that we follow rules that would yield the greatest happiness for the majority. Unlike act utilitarianism, rule utilitarianism often provides more practical guidance on actions. This is because it relies on pre-established general rules (e.g., respect for autonomy), while act utilitarianism requires on-the-spot calculations to determine what will maximise happiness in each unique situation (27). Third, *negative utilitarianism* also considers the consequences that affect well-being. It first focuses on what outcome minimises suffering and then what produces happiness for the largest number of people (28).

The utilitarian approach would base its ethical reasoning on a project based on the outcome. If the consequences of the outcome are for the common good, it can be argued to be ethically correct to proceed. However, a negative utilitarianism approach would first consider the amount of suffering the project provides and then what promotes the greatest happiness. For example, if a project based on statistical measures probably provides a better foundation for including CWD in policy or practice one can argue that it is ethically acceptable. On the other hand, if the project is likely to reinforce discrimination, it will not minimise suffering. Hence, it would be ethically challenging to proceed.

## Deontology

In contrast to utilitarianism, *deontology or Kantianism* does not determine what is right by its consequences. Deontology is the principle of following moral rules that define the right or wrong thing to do (29). In Immanuel Kant's deontology, it is essential to follow the *categorical imperative.* The categorical imperative implies that what one does in one situation should be the right thing to do in every situation. Applied to the categorisation of children as ‘CWD’, this would imply that the principles guiding how children are classified must be justifiable as universal rules, meaning that the same criteria should be applied consistently and fairly across cases. Therefore, Kant's categorical imperative is a universal command that applies to everyone, regardless of their circumstances or desires (29).

Further on, according to Kant, two important aspects of the categorical imperative are helpful. First, the action is only acceptable if its underlying principle, or the rationale behind an action (maxim), can be universally applied, also known as the *principle of universalisability* (27). To illustrate, if a researcher classifies children as ‘CWD’ using arbitrary or inconsistent criteria, the implicit maxim would be that such classification practices are acceptable. The maxim would be misconduct in research is acceptable’. In Kant's view, this would be wrong. A maxim is universalisable only if everyone can adopt it and achieve its objective (25). If misconduct is a maxim, research would become meaningless and hinder new truthful knowledge. Therefore, it is ethically and morally wrong.

The last principle, *principle of humanity,* tells us that we should treat individuals as ends in themselves, not merely as means to an end (29). In other words, research must respect individuals’ autonomy and treat them as rational human beings. One interpretation of Kantianism in a research context is that researchers must follow ethical guidelines, maintain openness, respect human individuals as meaningful subjects, and always be truthful. Therefore, researchers must treat the survey participants as subjective human beings, not as numbers and results. This also implies a distinction between conducting research on individuals and conducting research with them, emphasising the importance of recognising participants as active subjects rather than passive objects of analysis (30, 31).

## Virtue ethics

Virtue ethics, which emerged from Aristotle, emphasises character traits over moral rules. It advocates that virtuous individuals, through habitual right action, grow a characteristic disposition towards ethical behaviour (25, 32). According to a newer interpretation by Alasdair MacIntyre (32), virtue is not something we have, but we learn what a good virtue is through practice. In the context of research, virtues such as integrity, respect, and empathy are essential. It underscores the necessity of researchers not merely to ‘do’ ethics as a practical formality. Researchers must also ‘be’ ethical, and these virtues must be integrated into the professional identity (25, 33).

The virtue approach is particularly relevant in disability research, where the researcher's virtues directly impact the dignity and respect accorded to participants (25). For example, the virtue of empathy enables researchers to recognise the lived experiences of CWD, fostering a research environment that is inclusive and respectful. Additionally, virtue ethics complements other ethical frameworks. While deontology provides the ‘rules’ for ethical research and utilitarianism focuses on beneficial outcomes, virtue ethics offers a lens through which to view the researcher's character and intentions. This approach ensures a holistic and robust ethical framework. In conclusion, virtue ethics is fundamental in shaping ethical research practices. By fostering virtues, researchers can navigate the ethical landscape with a moral compass calibrated not just by rules or outcomes but by character and virtue.

## Application and discussion

This discussion examines the ethical implications of categorisation of CWD within quantitative research through the perspectives of utilitarianism, deontology, and virtue ethics. It is guided by core research principles such as *respect, good consequences, fairness, and integrity*, as reflected in international research ethics frameworks such as the Helsinki Declaration (34), as well as the National Committee for Research Ethics in the Social Sciences and the Humanities (7), which all Norwegian researchers ought to follow. Table 1 summarises the ethical framework.

**Table 1:** Comparison of ethical frameworks in disability research.

Ethical Framework	Focus	Justification for Categorisation	Potential Ethical Risks
Utilitarianism	Consequences and overall well-being	Categorisation is justified if it leads to better outcomes for the majority (e.g., improved policies or services)	May overlook individual rights or reinforce stigma if outcomes are deemed beneficial overall
Deontology	Moral duties and principles	Categorisation is acceptable only if it respects autonomy and treats individuals as ends, not means	Risk of violating personal dignity if categorisation is imposed without consent or clarity
Virtue ethics	Character and intentions of the researcher	Categorisation is justified when done with empathy, integrity, and ethical sensitivity	May lack clear guidelines; relies on researcher's moral judgment and virtues

From a *utilitarian* perspective, an ethical issue arises when trying to balance the potential benefits of the research with the potential harm or discomfort it might cause to the individuals involved (25). *Act utilitarianism* focuses on the consequences of individual actions, such as categorising disability, and weighs whether these actions lead to the greatest good for the greatest number. On the other hand, *rule utilitarianism* would consider whether the rules or guidelines followed in the research (such as categorising disability) lead to the most beneficial outcomes when followed consistently.

Lastly, *negative utilitarianism* would also consider the potential harm the project would have for the participants in a research project and other CWD. As mentioned earlier, both the medical and social understanding of disability will categorise children as CWD. The difference is that medical understanding uses a more objective measure, like diagnoses, whereas social understanding relies on more subjective measures (35). The latter understanding, as outlined in the CRPD (8), provides insight into how disability is constructed within the society in which individuals live, rather than by the individual themselves. Also, studies show that being part of a quantitative survey rarely has negative consequences for those involved (36). Hence, it would probably cause fewer negative consequences and promote better well-being. From a utilitarian standpoint, using `CWD` as a category in a quantitative study can be arguably appropriate, and it is what NESH (7) sees as seeking *good consequences*.

From a *deontological* perspective, the ethical issue involves respecting the rights and dignity of CWD while striving to conduct valuable and impactful research (25). As with the utilitarian perspective, the deontological perspective would argue that social understanding is better suited. This understanding underlines that we should not blame the individual human being but focus on society. If we apply the *principle of universality*, this would require that the methods used in the research are ones the researchers would want to see universally applied. If the data is collected using the recommended method for collecting disability data, for instance, from the WG (16), one can reasonably argue that the methods used are done according to the *principle of universality*.

The second principle, *principle of humanity*, tells us that we should treat individuals as ends in themselves (29). The individuals, CWD, must be treated as individuals with inherent dignity and worth, and not just numbers in a spreadsheet. Therefore, when categorising disability, it is important to consider whether the categorisation follows appropriate and justified principles within the field. For example, while the category ‘CWD’ is important and may be necessary for identifying inequality and supporting inclusion, researchers should be cautious not to treat this group as homogeneous. Rather, where relevant, distinctions can be made that reflect differences in types of disability, gender, age, levels of functioning, among others (2). At the same time, the continued use of the term ‘CWD’ can also be important, as it is used to recognise shared experiences and social barriers, and has increasingly been reclaimed as a term of identity and political recognition.

At the same time, using the label `CWD` grounded in a social understanding, may support a perspective in which disability is not attributed to the individual, but to societal barriers. Nevertheless, to ensure that the study follows the principle of humanity, the insight from intersectional theory (23) must be taken into account (24). This implies recognising that individuals are shaped by multiple, interacting social categories rather than a single defining characteristic. Using statistical approaches that acknowledge such within-group diversity may therefore help avoid overly simplified or essentialised categorisations. In this way, the risk of stigmatising labels can be reduced, and participants may be treated more respectfully.

From a deontological or Kantian perspective, one could reasonably argue that, as long as the categorisation process does not unfairly disadvantage certain groups, using the `CWD` category is ethical in research. However, the group must be treated with respect and dignity. Therefore, one must adopt a social understanding of disability and distance oneself from the medical understanding, which can be argued. The medical understanding does not respect the individual with a disability and advocates inequality and discrimination (37).

From a *virtue* ethics perspective, the ethical dilemma lies in embodying virtues such as honesty, integrity and courage in the research process (25, 38). When conducting research on vulnerable groups, it is essential for researchers to possess virtues such as respectfulness, humility, openness, and courage. In contrast to the two other approaches above, the virtue ethical approach focuses more on the researcher than the project (38). If the researchers in this project act according to virtues, they must evaluate whether the categories are done in a stigmatising or disrespectful way. For example, by using nuance and respectful language, the researcher will behave respectfully and be a fair researcher. In other words, be virtuous.

However, the virtues are not something the researcher possesses or lacks. The virtues must be learned by following a more competent person as a supervisor or expert (32). Researchers must navigate the challenge of conducting exact and valuable research while also being sensitive to the needs and experiences of CWD. The virtue standpoint might, therefore, argue that using labels can be seen as ethically wrong. Still, labelling can be ethically right if the researcher learns how to balance the virtues of openness, humility, and respect. In practice, this involves being transparent about the assumptions underlying categorisation, engaging critically with how categories are constructed, and ensuring that categories are applied consistently and respectfully throughout the research lifecycle, including in data collection, analysis, and reporting. From a virtue ethical standpoint, the right or wrong action lies more in what the researcher does than the outcome (32, 33). Making the research open and available for all promotes the obligation of the ethical principles that researchers should follow and respects and honours CWD.

## Concluding marks

This article has examined the ethical tension between the necessity of categorising children as `disabled` for statistical and policy purposes and the risk that such categorisation may reinforce discrimination and inequality. The analysis suggests that while this tension cannot be fully resolved, categorising can be ethically justified if applied with care, nuance, and respect for the individuals.

The findings highlight the importance of avoiding simplified and essentialist understandings of disability, while recognising that the category itself remains necessary for identifying inequality and informing policy. Ethical reflection should therefore be an ongoing process throughout the research lifecycle, rather than a one-time consideration.

## Data Availability

The original contributions presented in the study are included in the article/Supplementary Material, further inquiries can be directed to the corresponding author.
